# Brassinosteroid Signaling Pathways: Insights into Plant Responses under Abiotic Stress

**DOI:** 10.3390/ijms242417246

**Published:** 2023-12-08

**Authors:** Tanveer Alam Khan, Sajeesh Kappachery, Sameera Karumannil, Mohamed AlHosani, Nemah Almansoori, Hamda Almansoori, Mohammad Yusuf, Lam-Son Phan Tran, Mayank Anand Gururani

**Affiliations:** 1 Department of Biology, College of Science, United Arab Emirates University, Al Ain 15551, United Arab Emirates; tanveeralam@uaeu.ac.ae (T.A.K.); sajeesh_k@uaeu.ac.ae (S.K.); sameerashameem@gmail.com (S.K.); 201711661@uaeu.ac.ae (M.A.); 201814153@uaeu.ac.ae (N.A.); 201814795@uaeu.ac.ae (H.A.); myusuf.alig@uaeu.ac.ae (M.Y.); 2 Institute of Genomics for Crop Abiotic Stress Tolerance, Department of Plant and Soil Science, Texas Tech University, Lubbock, TX 79409, USA

**Keywords:** abiotic stress, abscisic acid, brassinosteroids, ethylene, hydrogen peroxide, signaling pathway

## Abstract

With the growing global population, abiotic factors have emerged as a formidable threat to agricultural food production. If left unaddressed, these stress factors might reduce food yields by up to 25% by 2050. Plants utilize natural mechanisms, such as reactive oxygen species scavenging, to mitigate the adverse impacts of abiotic stressors. Diverse plants exhibit unique adaptations to abiotic stresses, which are regulated by phytohormones at various levels. Brassinosteroids (BRs) play a crucial role in controlling essential physiological processes in plants, including seed germination, xylem differentiation, and reproduction. The BR cascade serves as the mechanism through which plants respond to environmental stimuli, including drought and extreme temperatures. Despite two decades of research, the complex signaling of BRs under different stress conditions is still being elucidated. Manipulating BR signaling, biosynthesis, or perception holds promise for enhancing crop resilience. This review explores the role of BRs in signaling cascades and summarizes their substantial contribution to plants’ ability to withstand abiotic stresses.

## 1. Introduction

**Figure 1:**
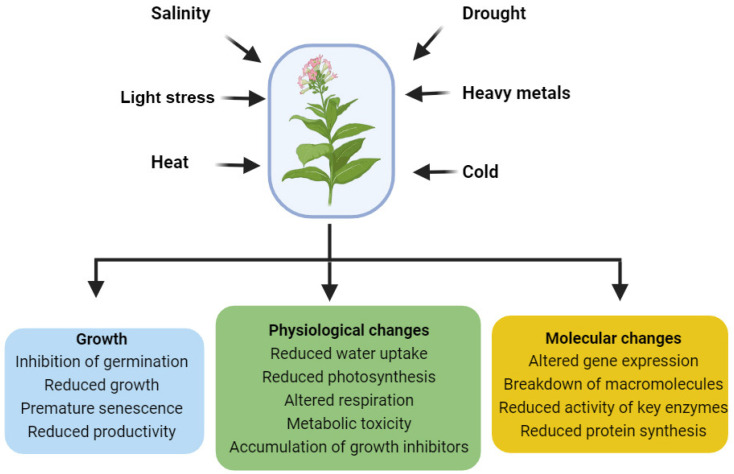
Abiotic stresses affect different aspects of plant growth and development. This figure was drawn using BioRender [https://www.biorender.com (accessed on 20 August 2023)].

The ability of plants to withstand abiotic stresses is enhanced through internal defense mechanisms [1]. Abiotic stresses encompass nonliving environmental conditions detrimental to plant development, eliciting diverse physiological, molecular, and growth responses in plants (Figure 1). These responses include gene regulation adjustments, altered protein and metabolite production, hormone signaling changes, and increased antioxidant enzyme activities [2]. Phytohormones, which are naturally occurring organic compounds, exert a substantial influence on crucial plant life cycle processes at low concentrations [3]. Their involvement in signal transduction networks enhances plant development and productivity in response to abiotic challenges [4]. Brassinosteroids (BRs) regulate a variety of biological and cellular processes, including stem elongation, pollen tube development, leaf morphology, root growth inhibition, fruit maturation, ethylene production, xylem separation, chlorophyll production, and gene expression [5,6]. Furthermore, BRs have been demonstrated to modify antioxidant enzyme and nonenzymatic defense mechanisms as well as enhancing plant development and chlorophyll, sugar, and proline content [7,8]. Previous studies have highlighted BRs’ positive impact on *Solanum nigrum* L., enhancing sugar and photosynthetic pigment accumulation, improving photosystem II (PSII) efficiency, and reducing electrolyte leakage, malondialdehyde levels, and cadmium (Cd) accumulation [9]. Additionally, BRs enhance rice growth and biomass under chromium (Cr) stress, influencing nutrient uptake and modulating antioxidant enzyme activity [7,9]. Furthermore, BRs elevate enzymatic activity and mitigate oxidative damage in *Raphanus sativus* L. during extreme reaction oxygen species (ROS) levels [10]. Similarly, exogenous BR administration has been shown to increase rice biomass and overall growth [7]. BRs also mitigate abiotic stressors, such high temperature, chilling stress, and metal stress [11,12,13,14]. Considering their crucial role in protecting plants from environmental stresses, BRs also play a pivotal role in sustainable crop production [8]. Therefore, adjusting BR signaling, biosynthesis routes, or perceptions holds promise for crop improvement. This review aims to explore the genuine role of BRs in signal cascades and outline their beneficial contributions to abiotic stress resistance.

## 2. Structure and Biosynthesis of BRs

**Figure 2:**
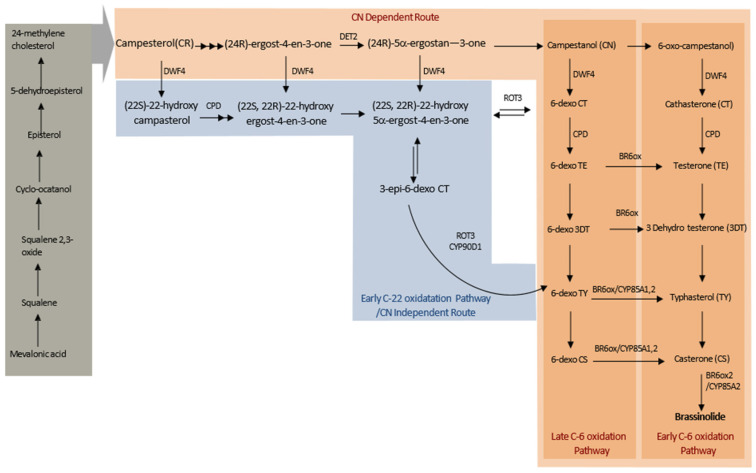
Biosynthetic pathways of BR. Different colours represent different pathways.

BRs are a class of polyhydroxylated steroidal phytohormones that are found naturally in plants and are essential to their regular growth and development. Depending on their C-24 alkyl substituents, these steroids are classified as C27, C28, or C29 [15]. Brassinolide (BL), the most physiologically active chemical of all the BRs discovered so far, is present in a wide variety of plant species [16]. The primary emphasis of BR research is on BL, a 28-carbon molecule with an S-methyl group at position C24 of the side chain of the 5α-ergastane structure. According to Zhao and Li [17], other BRs are primarily inactivated metabolites that come from different catabolic processes of BRs or intermediates of the BL biosynthesis pathway. A detailed analysis of the biosynthesis of BL, a C28 BR, showed that the early and late C-6 oxidation processes operate in tandem [18]. Campesterol (CR), the biosynthetic precursor specific to BR, is first transformed to campestanol (CN), and then to BL by early or late C-6 oxidation routes. Since the precursor, CN, is the starting point for both the early and late C-6 oxidation processes, they are referred to as CN-dependent pathways. In crop plants like tomato and tobacco, the late C-6 oxidation pathway appears to be the predominant route because most of the endogenous BRs in these species comprise only members of the late C-6 oxidation pathway. In the early C-6 oxidation pathway, C-6 oxidation takes place before DWF4-mediated C-22 hydroxylation. In the early C-6 oxidation pathway, CN is mainly converted to 6-oxocampestanol (6-oxoCN) and then to cathasterone (CT), teasterone (TE), 3-dehydroteaserone (3DT), typhasterol (TY), and then to castasterone (CS), in order. In the late C-6 oxidation pathway, C-22 hydroxylation takes place ahead of C-6 oxidation. The intemediates undergo further modification and are included into the late C-6 oxidation pathway following CR’s hydroxylation by DWF4. To create 6-deoxocathasterone (6-deoxoCT), CN is first hydroxylated at C-22. It is subsequently transformed to matching intermediates, like those in the early C-6 oxidation pathway, but in a C-6 deoxy form [17]. The enzymes CYP85A1 and CYP85A2, respectively, catalyse the oxidation processes of 6-deoxoTY and 6-deoxoCS into TY and CS [16]. On the other hand, BR6ox connects the early and late C-6 oxidation pathways in *Arabidopsis* at many locations. Additionally, DWF4 is CN-independent since it can operate on several biosynthetic intermediates in the upstream pathways. An early C-22 hydroxylation pathway can be established by the pathways branching at campesterol [15]. Figure 2 depicts the synopsis of the BRs biosynthesis pathway.

## 3. Role of BRs in Plant Growth and Development 

BRs function as steroid hormones, influencing plant growth and development [19,20]. These compounds are implicated in diverse biological processes in addition to plant growth, including stem cell maintenance, cell division, vascular growth, and flowering [21,22]. Hydroponically grown plants can leverage BRs to stimulate growth through activation of the cell cycle during seed sprouting, cell cycle control, and leaf growth. BRs play a vital role in regulating responses to both abiotic and biotic stresses as well as the formation of stomata [20,23]. Furthermore, BRs govern the fertility of both female and male crops [24]. BRs influence etiolation, promote stigma elongation [25] and influence leaf size and angle, responses to atmospheric pollution, and thermotolerance [26,27]. Exogenous BR application or manipulation of BR biosynthesis and signaling pathways has the potential to enhance crop yields [28]. Chen et al. [25] discovered that the regulation of *Arabidopsis thaliana* L. growth is regulated by the BR-activated *WRKY46*, *WRKY54*, and *WRKY70*. In addition, the histone lysine methyltransferase SDG8 emerged a critical regulator of BR-regulated gene expression, with a knockout mutant exhibiting impaired BR response and reduced growth in *Arabidopsis* [29].

In *Arabidopsis*, BR also modulates transcriptional pathways controlling seed and ovule development, influencing their size, weight, and number [30]. BRs also regulate the response to mild nitrogen deficits in *Arabidopsis*, mediating the elongation of primary roots through the *BSK3* gene [31]. In rice, BR controls plant structure and grain yield, with BRD1 and D11, which influence plant height, implicated in BR biosynthesis [32]. OsDwarf2/OsDwarf1, enzymes that contribute to BR biosynthesis, are known to negatively affect second internode and seed size in rice [32]. During rice panicle development, antioxidant system activation and energy charge are elevated, promoting spikelet growth in response to nitrogen fertilization [33]. In wheat, exogenous BR application delays the transition from vegetative to generative states, whereas the BR inhibitor brassinazole accelerates the transition and heading steps [34].

Winter rapeseed matures 4–8 days faster with BR application [31], and interactions between BRs and other plant hormones enhance plant performance and adjust growth [35]. Disruption of BR signaling impacts seed formation, pollen development, flowering duration, and other developmental processes [36]. BR-deficient plants exhibit notable characteristics such as short hypocotyls, petioles, and internodes as well as downward-curled leaves, delayed flowering, altered stomatal development, lower male fertility, and reduced plant structure due to decreased lamina inclination [36,37]. According to Zhu et al. [38], BR deficiency results in smaller grains, less fertile seeds, fewer tillers, inappropriate stomatal distribution, and decreased seed germination. BR-insensitive or BR-deficient mutants are often referred to as late-flowering mutants owing to their slow growth [17]. Conversely, plants accumulating excess BRs display enlarged petioles and hypocotyls, resulting in increased height [39].

## 4. BRs and Redox Homeostasis

A high-rate redox metabolic process like photosynthesis is sensitive to abrupt changes in its input factors. ROS, including singlet oxygen, superoxide anion radicals, and H_2_O_2_, are generated during photosynthesis because of quick fluctuations in photon capture, electron fluxes, and redox potentials. As a result, the photosynthesizing chloroplast serves as a conditional source of crucial redox and ROS that is used to adjust processes within the chloroplast as well as in the cytosol and nucleus after retrograde release or processing. However, signaling proteins may be directly oxidized and modulated by ROS. H_2_O_2_ and the GSH:GSSG ratio did not significantly rise at very low BR concentrations. In cucumber plants, there was a notable rise in both the H_2_O_2_ content and the GSH:GSSG ratio upon increasing BR. According to Jiang et al. [40], there is a possibility that markedly increased H_2_O_2_ will serve as a signal for a greater decrease in GSSG to GSH, most likely due to increased GR activity. H_2_O_2_ concentrations rose in response to a rise in BR, which may have led to oxidative stress. As a part of their cellular antioxidative reactions, plants enhance their synthesis of glutathione in such circumstances, as seen by the elevated GSH+GSSG concentration. Under high ROS concentrations, the ratio of GSH:GSSG also dropped, most likely because of enhanced GSH oxidation for the scavenging of ROS or other harmful chemicals. Because of this, the cellular redox state’s reaction to BR and H_2_O_2_ showed different phases and, as a result, the beneficial effects of both BR and H_2_O_2_ on CO_2_ assimilation were only shown in cucumbers at moderate concentrations [40].

## 5. BRs as Regulators of Abiotic Stress Responses

For many years, researchers have investigated the impact of abiotic stress on phytohormone levels and their signaling status. These changes reveal that growth regulators act as mediators of various upstream signals rather than as early transducers of stress signals. With the aim of meeting the evolving needs of plants, this review explores the effects of BRs, as a class of environmentally safe hormones, on crop responses. Using BRs could facilitate a marked improvement in plants’ abilities to withstand stress, resulting in improved quality and increased yield. Numerous studies conducted over several years have suggested the involvement of BRs and related chemicals in plant responses to diverse abiotic stresses. Table 1 summarizes the current findings regarding the role of BRs in regulating such responses in plants.

## 6. BRs and Drought

Drought, resulting from insufficient rain or water, drastically reduces crop yield, affecting various physiological processes through absorption, extrusion, retention, and osmotic stress, which disturb redox balance [48]. Drought resistance is associated with abscisic acid (ABA) accumulation. Exogenous BR treatment elevates ABA levels, mitigating the adverse effects of drought on plants. In challenging environments, BR dosage in tomatoes promotes drought resistance by improving photosynthetic machinery, leaf hydration, and antioxidant defense [49]. Khamsuk et al. [50] revealed that exogenous BRs enhance light use and stimulation intensity in pepper seedlings during drought, thus increasing resistance to drought-induced dehydration through foliar application [50]. The long-term consequences of drought can be alleviated with BR interventions [51]. Despite drought inducing excessive amounts of ROS, the presence of BRs significantly reduces ROS, malondialdehyde, and lipid peroxidation levels [52].

Exogenous BR administration promotes drought resistance, as observed in mutants with increased stress tolerance, including both BR-deficient and BR-insensitive mutants [53]. In tomatoes, increased BR levels, rather than BR signaling potency, enhance drought tolerance; whereas, *BRI1* overexpression negatively impacts tomato drought tolerance, revealing the dual role of the BR network in stress tolerance [54]. Furthermore, BRs and ABA are involved in mostly antagonistic physiological processes [52,53]. BIN2 inhibits BR signaling, whereas ABA-mediated stress responses are enhanced by *SnRK2* phosphorylation, which activates ABA-sensitive genes [55]. Exogenous BR inhibits ABA-induced transcriptional activation of RD26, a transcriptional activator of stress-induced genes [56]. Reciprocal antagonistic interactions between BR signaling and ABA-responsive transcription factors in *Arabidopsis* contribute to plant development and drought tolerance. RD26 targets *BES1*, allowing for BR to suppress RD26 under drought conditions, modulating transcription of BES1-controlled genes, consequently diminishing BR function [57].

**Figure 3:**
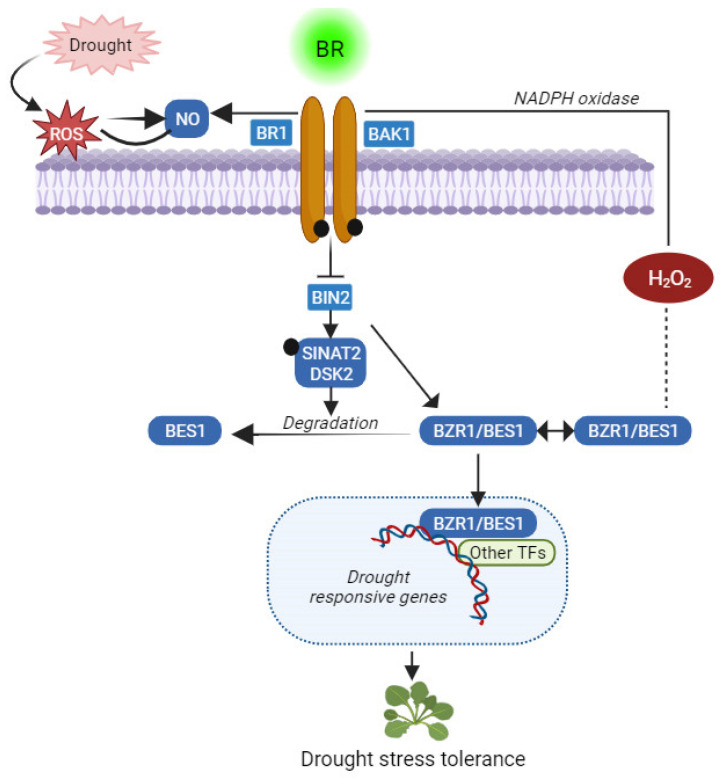
BRs control the plant development–stress response equilibrium. Depending on cellular and environmental settings, interactions between BR and stress signaling pathways can occur through their receptors, downstream kinases (such as BIN2), and/or transcription factors (such as BZR1/BES1). This figure was drawn using BioRender [https://www.biorender.com accessed on 29 September 2023)].

Research suggests that autophagy pathways, interacting with BR signaling via *BIN2*, regulate drought stress and malnutrition [58] (Figure 3). BIN2 activates a phosphorylated DSK2 ubiquitin receptor protein, directing it to degrade BES1 through autophagy [58]. Collectively, these findings highlight the complexity of BR-mediated drought responses.

## 7. BRs in Plant Response to Extreme Temperature Stresses

Chilling or freezing damage from low temperatures notably impedes the production of agricultural products worldwide, particularly affecting thermophilic plants [59]. Under cold stress, plants face mechanical constraints, membrane fluidity differences, macromolecule interactions, and osmotic pressure changes. Cold exposure adversely impacts plant photosynthetic activity, leading to decreased CO_2_ assimilation, photoinhibition of PSI and PSII, and reduced enzyme activity [60].

Enhanced cold tolerance, induced by BRs, affects not only entire plants but also the quality of harvested products, such as fruit. Postharvest management requires comparatively larger amounts of BRs relative to those needed for stress responses across the entire plant. Cold stress compromises the integrity of tomato fruit, whereas mango fruit exhibits increased concentrations of protective proteins, including remorin and temperature-induced lipocalin, which protect the fruit against cold-related damage [61]. Additionally, BRs reduce phase transition temperatures and increase fluidity under cold conditions by augmenting unsaturated fatty acids in the phospholipids of the mango fruit plasma membranes [61]. Various BR levels influence the freshness of peppers at low temperatures. Raza et al. [62] found that BRs effectively mitigated chilling damage in green sweet peppers, enhancing antioxidant activity, photosynthetic pigments, and L-ascorbic acid levels. It was hypothesized that this would lessen oxidative damage and electrolyte leakage during cold stress.

**Figure 4:**
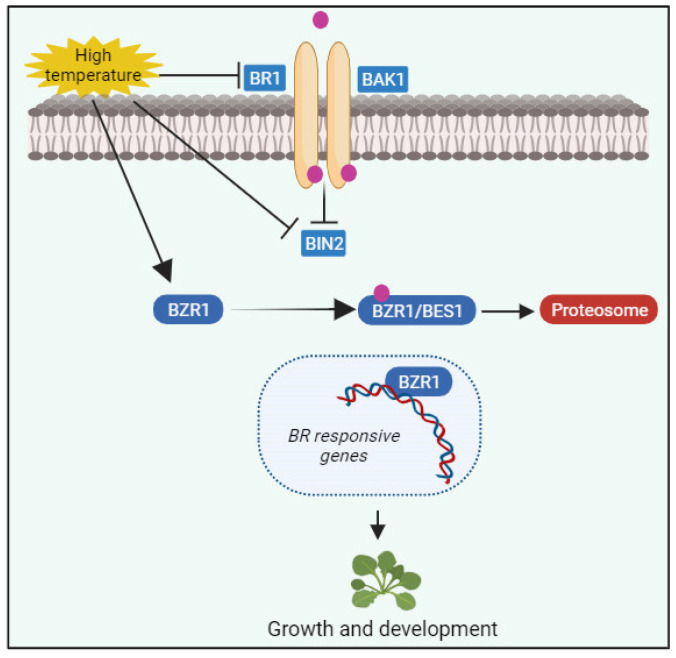
BR signaling amid high-temperature stress and growth responses. BZR1 accumulation in the nucleus during an increase in temperature regulates thermomorphogenesis by inducing gene expression that promotes growth or binding to PIF4 promoters. High temperatures lead to active PIF4 accumulation, favouring BES1–PIF heterodimers in nuclear protein complexes rather than BES1 homodimers. High concentrations of BES1–PIF4 centres activate thermomorphogenesis-related genes. This figure was drawn using BioRender [(https://www.biorender.com (accessed on 15 September 2023)].

Regarding heat stress, in terms of physiological activity, the photosynthetic apparatus is particularly sensitive. Moreover, high temperatures markedly affect the connection between PSII and photochemical activities, resulting in decreased net photosynthetic rates [63]. BR pretreatment in tomato plants reduces heat-induced losses in photosynthesis by enhancing antioxidant enzyme activity, thereby mitigating oxidative damage under stress. Intriguingly, BRs play a role in regulating thermotolerance in both heat-tolerant and heat-sensitive plant cultivars [64]. For instance, BR pretreatment significantly enhances photosynthetic rate, net CO_2_ absorption rate, stomatal closure, PSI photodegradation activity, and water use efficiency in both heat-tolerant and heat-sensitive melons [62]. In eggplant, BRs boost antioxidant capacity, reducing ROS accumulation and alleviating heat stress [65]. BR signaling also regulates plant growth under high-temperature stress (Figure 4). BZR1 accumulation in the nucleus during an increase in temperature regulates thermomorphogenesis by inducing gene expression that promotes growth or binding to PIF4 promoters [66]. High temperatures lead to active PIF4 accumulation, favoring BES1–PIF heterodimers in nuclear protein complexes rather than BES1 homodimers [67]. This reduces the available active BES1 homodimers, depressing BR biosynthesis and inhibiting BR signaling. High concentrations of BES1–PIF4 centers activate thermomorphogenesis-related genes [67]. Moreover, the kinase-defective BRI1 protein from bri1-301 mutants exhibits decreased stability and biochemical activity under high temperature, suggesting temperature-enhanced misfolding and degradation of this protein in the mutant [68]. Thus, these studies reveal the involvement of BR receptors and downstream signaling elements in regulating growth responses to temperature changes.

## 8. Interaction of BRs with Other Hormonal Pathways

Plants respond to various environmental cues and developmental signals through crosstalk between the BR pathway and other hormonal pathways. Both plant hormones and BRs play pivotal roles in regulating plant development, growth, and stress responses. In the following sections, examples illustrating the crosstalk between BRs and other hormonal pathways are provided. 

### 8.1. BRs and Ethylene

BRs and ethylene, as vital plant hormones, orchestrate various aspects of plant development and responses to environmental signals. Despite primarily using distinct signaling pathways, evidence suggests that crosstalk between these two hormone pathways coordinates plant responses and optimizes growth under changing conditions. Ethylene, a fundamental gaseous plant hormone produced by nearly all plant tissues, influences critical physiological processes and stress responses. A previous study showed that BRs impede stem elongation, increase lateral expansion, and exacerbate the apical hook curve in *Pisum sativum* L. seedlings exposed to BRs [69]. Additionally, BRs were found to boost endogenous ethylene synthesis, with ethylene mediating BRs’ inhibitory impact [69]. In *Solanum lycopersicum* L., the fruit of SlCYP90B3-OE transgenic lines exhibited higher ethylene content compared with control fruit, demonstrating increased ethylene production through the upregulation of ethylene biosynthesis genes (*SlACS2*, *SlACS4*, and *SlACO1*) and signaling genes (*SIETR3* and *SICTR1*) [70].

BRs contribute to the ethylene biosynthetic pathway by controlling signaling and ethylene biosynthesis-related genes, For example, the expression of ACO_2_ and endogenous ethylene levels in *Arabidopsis thaliana* L. are controlled through the BR transcriptional component BES1 [71]. Alternative oxidase (AOX) may be employed to protect photosystems from BL-mediated ROS accumulation in *Nicotiana benthamiana* L., enhancing the plants’ ability to withstand abiotic stress [72]. BL, a BR, increases ethylene production and AOX expression in *Cucumis sativus* L. seedlings subjected to dehydration, salt, and freezing stresses [73]. Ethylene was shown to restore BL-induced negative effects; however, pretreatment with aminooxy acetic acid, which inhibits ethylene biosynthesis, markedly reduced seedling resistance to BL-induced photooxidation [73]. BR pretreatment increases ethylene production and signaling during salt stress by enhancing 1-aminocyclopropane-1-carboxylate synthase activity, an enzyme implicated in ethylene synthesis [74]. However, limiting ethylene production and/or signaling elements reduces salt sensitivity and antioxidant enzyme activity produced by BR [74].

BRI1, a membrane-bound BR signaling center, may play a role in regulating salt stress tolerance, as indicated by Bri1-9 mutants’ partial recovery from salt hypersensitivity when their endoplasmic reticulum-associated protein degradation pathway is suppressed [75]. Bin2-1 mutants, characterized by the activation of fewer stress-responsive genes, exhibit heightened sensitivity to salt stress [76]. High salinity promotes root growth quiescence by preventing *BZR1* nuclear accumulation and subsequent BR signaling functions [77].

**Figure 5:**
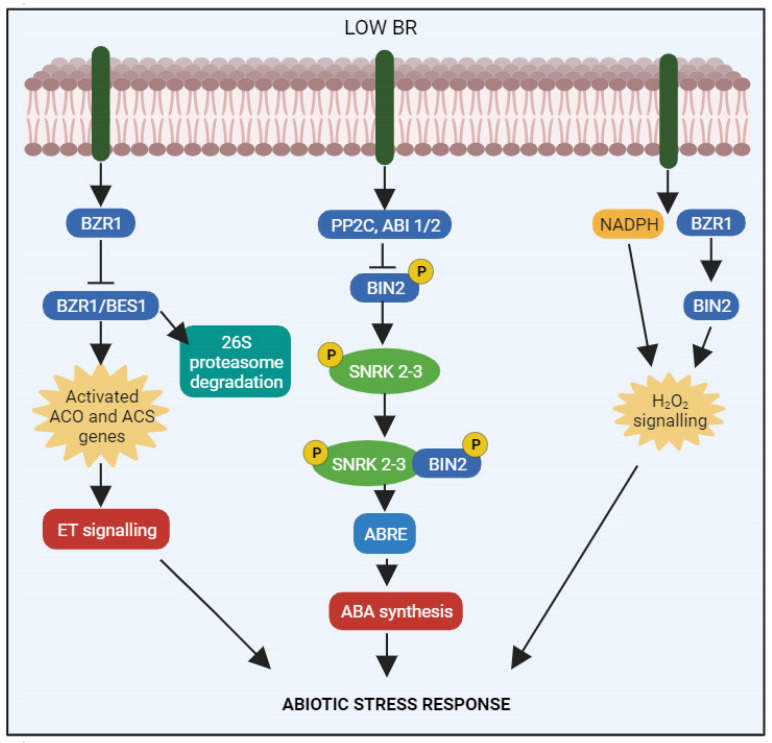
Interactions of BRs with other hormonal pathways. ET: BRs promote the expression of 1-aminocyclopropane-1-carboxylic acid oxidase (ACO) and 1-aminocyclopropane-1-carboxylic acid synthase (ACS) to synthesize ethylene. BRs control the transcriptional and post-transcriptional regulation of ethylene production by lengthening the half-life of the ACS protein. Ethylene biosynthesis is either negatively or positively controlled by BR, depending on the dose. BR accelerates the expression of genes associated with ethylene production, including ACS. Moreover, BR stabilizes ACS proteins by breaking down 26S proteasomes and promotes the post-transcriptional production of ACS. ABA: By encouraging the phosphorylation and activation of SnRKs, ABA is recognized by PYR/PYL/RCAR receptors and releases SnRKs from PP2C-mediated repression. Subsequently, SnRKs phosphorylate downstream transcription factors, like ABI1/2, which control the transcription of several genes that respond to stress. In addition to directly phosphorylating and activating SnRKs and ABI1/2, BIN2, a negative regulator of BR signalling, can also be inactivated by PP2C BZR1 and also directly targets ABI, suppressing its transcription to adversely affect the expression of stress-responsive genes. H_2_O_2_: BR-encouraged H_2_O_2_ levels were initiated by an NADPH-dependent pathway. When BR binds to receptor kinase BRI1, it not only raises the cellular level of H_2_O_2_, which oxidizes BZR1 at a conserved cysteine residue, but also suppresses the kinase activity of BIN2 to induce dephosphorylation of BZR1. The transcriptional activity of BZR1 is enhanced by this oxidation. ET= ethylene; ABA= abscisic acid; H_2_O_2_ = hydrogen peroxide, PP2C = protein phosphatase 2C; ABI = abscisic acid-insensitive; SNRK = SNF1-regulated protein kinase; ABRE = abscisic acid responsive element. This figure was drawn using BioRender [(https://www.biorender.com (accessed on 26 August 2023)].

Overall, ethylene-BR crosstalk fine-tunes plant development and responses to diverse environmental inputs; however, the molecular mechanisms underlying this crosstalk are complex and remain the subjects of ongoing research. Various interactions can affect the plant, its growth stage, and its environment. BRs promote the expression of 1-aminocyclopropane-1-carboxylic acid oxidase and 1-aminocyclopropane-1-carboxylic acid to synthesize ethylene (Figure 5). BRs control the transcriptional and post-transcriptional regulation of ethylene production by lengthening the half-life of the ACS5 protein [78]. Ethylene biosynthesis is either negatively or positively controlled by BR, depending on the dose [21]. Exogenous BR application accelerates banana ripening by regulating the expression of genes associated with ethylene production, including *MaACS1*, *MaACO13*, and *MaACO14* [79]. In tomato plants, BR increases the post-transcriptional expression of ACS2 and ACS4 [80]. In addition to ACS6, ACS9, and ACS5, BR stabilizes other ACS proteins by degrading 26S proteasomes [78]. Indirect evidence suggests that BR increases ethylene synthesis by regulating the *ROT3/CYP90C1* gene, which in turn controls hyponastic growth [81].

### 8.2. BRs and Hydrogen Peroxide

The interplay between BRs and hydrogen peroxide (H_2_O_2_) is essential for numerous physiological processes and responses to environmental stimuli. Investigating this crosstalk offers valuable insights into plant growth, development, and stress responses. H_2_O_2_, an ROS, serves as a signaling molecule in plants [82]. It is produced in response to various environmental cues and stresses, such as pathogen attacks, drought, high light intensity, and mechanical damage. Serving as a secondary messenger, H_2_O_2_ regulates crucial processes, including the cell cycle, apoptosis, and stress adaptation. A previous study revealed that introducing H_2_O_2_ to tomato plants during drought stress increased root respiration, chlorophyll levels, and yield [83]. Although high concentrations of H_2_O_2_ can lead to an oxidative burst that damages protein structures and impairs cell signaling, low concentrations may enhance plant tolerance to stress induced by high temperature [13], low temperature [12], copper exposure, and heavy metal toxicity [84]. A study on *Cucumis sativus* L. revealed interactions between BRs and H_2_O_2_ in relation to sugar uptake and the Calvin cycle, indicating that H_2_O_2_ controls photosynthesis via BRs [40]. Another study demonstrated that BR treatment significantly elevated H_2_O_2_ levels in *A. thaliana* L. seedlings through an NADPH-dependent mechanism, thereby influencing seedling development [85]. The study also showed that diphenylene iodonium treatment significantly reduced the effects of BRs on hypocotyl elongation and significantly lowered H_2_O_2_ levels.

By affecting BRs and H_2_O_2_, when combined, they show promising effects in enhancing crop productivity through their impact on photosynthesis and sugar metabolism. In response to cold stress, *Lycopersicon esculentum* L. exhibited increased SPAD chlorophyll levels, net photosynthetic rate, carbonic anhydrase activity, and other antioxidant enzyme activities [12]. Nazir et al. [14] explored the possibility of reducing Cu toxicity in *Lycopersicon esculentum* L. through combined BR and H_2_O_2_ treatment, finding that this combination considerably improved chlorophyll levels and the Fv/Fm ratio compared with individual treatments. BRs and H_2_O_2_ reduced electrolyte leakage while increasing net photosynthetic rate and associated attributes. The interaction also impacted total protein content, antioxidant enzyme activities, and carbonic anhydrase activity as well as chloroplast ultrastructure and stomatal performance in Cu-treated tomato seedlings. Therefore, the BR–H_2_O_2_ interaction may improve total protein content and photosynthetic capacity while sustaining the antioxidant system and plasma membrane, thereby enhancing plants’ ability to withstand abiotic stress. Additionally, the BR–H_2_O_2_ interaction has been shown to elevate lycopene and β-carotene levels in fruit [12]. Furthermore, BRs and/or H_2_O_2_ have been shown to improve cell water relations, e.g., membrane stability and leaf water potential, and reduce electrolyte leakage, contributing to the maintenance of normal cellular metabolism [12,86]. Tian et al. [85] demonstrated that BR treatment dramatically increased the H_2_O_2_ content in *Arabidopsis thaliana* L. seedlings, and that BR-encouraged H_2_O_2_ levels were initiated by an NADPH-dependent pathway (Figure 5). Then, they looked at any potential involvement of H_2_O_2_ in BR-facilitated seedling growth. They demonstrated that treatment with diphenylene iodonium (DPI), an inhibitor of NADPH oxidase, reduced H_2_O_2_ levels and hypocotyl elongation was greatly reduced by BRs. High DPI concentrations, however, made *Arabidopsis thaliana* L. seedlings less sensitive to BRs [85]. 

In summary, the complex crosstalk between BRs and H_2_O_2_ in plants integrates hormonal and oxidative signals to regulate diverse physiological processes. This interaction serves as a mechanism for plants to optimize growth, development, and responses to environmental stresses, ultimately enhancing their adaptability and survival.

### 8.3. BRs and Abscisic Acid

Plants use abscisic acid (ABA) as a stress sensor to combat abiotic stresses [87]. In response to salt stress, plants regulate ABA levels to mitigate its effects. Rapid ABA accumulation leads to swift closure of stomata, reducing transpirational water loss [88]. Leaf tissues with elevated ABA concentrations facilitate salt adaptation by modulating stomatal activity, adjusting osmotic levels, and increasing stress protein production [88]. ABA significantly improves freezing, chilling, drought, and salt tolerance in a various plants [89]. Heavy metals, such as aluminum, zinc, cadmium, and nickel, have been shown to elevate ABA levels in plants [90]. Among these, Cd, a hazardous divalent heavy metal, is rapidly absorbed by bacteria, inducing detrimental symptoms, such as stunted growth. [91]. Cd also adversely affects plant photosynthesis by lowering chlorophyll levels and inhibiting stomatal opening [92]. In *Phragmites* and *Typha* plants, Cd-induced ABA accumulates in roots but not shoots [90]. 

Although ABA and BRs generally have distinct functions, evidence suggests that they can interact and crosstalk in specific physiological processes. ABA slows seed sprouting and improves seed dormancy through embryo development, whereas BR accelerates seed sprouting [93]. Furthermore, BR and ABA play contrasting roles in modulating seed germination and post-germinative development [92]. Hussain et al. [94] found that the exogenous administration of ABA, BRs, and ABA + BRs enhanced agronomic indicators and photosynthetic qualities in rice plants exposed to varying levels of salt stress. Additionally, exogenous hormone treatment improved pollen viability, spikelet source-to-sink capacity, and leaf area as well as net photosynthetic rate and SPAD values, in rice flag leaves. Additionally, the joint application of BRs and ABA increased grain weight under salt stress in rice [94].

The interaction between BR and ABA involves gene expression regulation and protein activity modulation. In the presence of BR, a complex involving topless (TPL/TPR), BRI1-EMS suppressor 1, and histone deacetylase 19 suppresses the expression of the gene *ABI3* through its effects on the E-box promoter. *BZR1* transcription factor binding to the *ABI5* G-Box promoter regions suppresses *ABI3* and *ABI5* gene expression, mitigating the stress response (Figure 5). At low BR levels, BIN2 increases *SnRK2/3* activation, triggering stress responses. *BIN2* phosphorylates the *ABI5* transcription factor, upregulating the expression of genes associated with ABA [93]. In some studies, the autostimulation of ABA-associated *SnRK2s* genes and kinase activity was observed [95,96]. Researchers are actively exploring the molecular mechanisms underlying this crosstalk to deepen our understanding of how plants integrate diverse hormonal signals for optimized growth and adaptive responses to changing environments.

## 9. BR Signaling Pathway

The molecular and metabolic aspects of BR signaling in plants have been extensively investigated. The importance of the BR hormone signalling pathway in growth control and the regulation of many genes related to the cell wall are highlighted, as is the discovery of a receptor-like protein (RLP44) that is essential for triggering BR signalling through direct interaction with the BR coreceptor BAK1. This interplay combines hormone signalling and cell wall surveillance to maintain cell wall integrity and homeostasis, which in turn affects plant development [97]. However, the BR signal transduction pathway in *Arabidopsis thaliana* L. starts with ligand detection on the cell membrane and ends with gene expression in the nucleus. Upon BR binding to the plasma membrane-anchored leucine rich repeat receptor-like kinase (BRI1) receptor, a signal cascade activates the expression of genes through nuclear and cytosolic transcription kinases and phosphatases [98]. *BRI1* enhances its kinase activity through successive transphosphorylation with *BAK1* and autophosphorylating multiple times upon BR detection. In this process, *BRI1* promptly releases *BKI1*, a negative regulator at the C-terminus [99]. *BKI1* improves BRs signaling by degrading 14-3-3 proteins, which maintain the cytoplasmic retention of two master transcription factors: *BZR1* and *BES1* [100,101]. *BRI1* activation induces the activation of *BSU1* phosphatase through the phosphorylation of BSKs. Activated *BSU1*, in turn, dephosphorylates *BIN2*, rendering it inactive [102].

**Figure 6:**
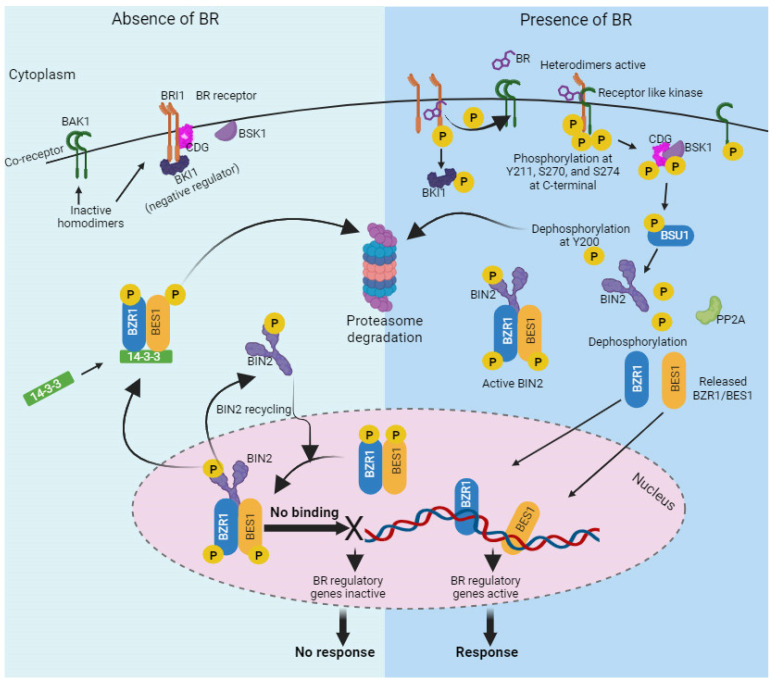
BR signalling pathway. The perception of BR begins at the plasma membrane. The BR receptor complex consists of the receptor kinase BRASSINOSTEROID-INSENSITIVE 1 (*BRI1*) and its co-receptor *BRI1*-ASSOCIATED RECEPTOR KINASE 1 (*BAK1*). When BRs bind to the BRI1 receptor, they induce a conformational change in the receptor, leading to the activation of its kinase domain. Activated *BRI1* phosphorylates itself and phosphorylates *BAK1*. The activated *BRI1-BAK1* complex initiates a phosphorylation cascade involving several downstream components, including BR-SIGNALING KINASE 1 (*BSK1*), CONSTITUTIVE DIFFERENTIAL GROWTH 1 (*CDG1*), and others. *BIN2* (BRASSINOSTEROID-INSENSITIVE 2), a *GSK3*-like kinase, is a negative regulator of the BR signalling pathway. Activated *BRI1* inhibits *BIN2* through phosphorylation. Inhibition of *BIN2* leads to the stabilization and nuclear translocation of *BZR1* (BRASSINAZOLE-RESISTANT 1) and *BES1* (*BRI1*-EMS-SUPPRESSOR 1), which are transcription factors that regulate the expression of BR-responsive genes. *BZR1* and *BES1* bind to specific DNA sequences in the promoters of target genes, regulating their expression. This figure was drawn using BioRender [https://www.biorender.com (accessed on 28 August 2023)].

The master transcription factors *BZR1* and *BES1* are released from suppression due to *BIN2* inactivation. Activated *BZR1* and *BES1* translocate into the nucleus to directly control the expression of BR-related genes or interact with other transcription factors [103]. *BKI1* maintains BRI1 in an inactive state when BRs are absent [101,104]. Both *BZR1* and *BZR2*/*BES1* are nuclear transcription factors that are phosphorylated by the *GSK3* kinase repressor protein *BIN2*, present throughout the nucleus, cytoplasm, and plasma membrane. Consequently, the activity of these two transcription factors is restricted. *BIN2* prevents *BZR1* and *BZR2/BES1* from becoming functional transcription factors by impeding their interaction with other proteins or transcription factors [105]. Figure 6 depicts the BR signaling scheme.

## 10. Conclusions

BRs are considered pivotal in abiotic stress responses as they regulate a specific set of genes to mediate abiotic stress tolerance responses. BRs regulate the transcription of these genes, encoding essential proteins and enzymes, thereby safeguarding plants and preventing them from succumbing to stress. Notably, BRs and related compounds have been implicated in stabilizing responses to various abiotic stimuli, including drought and temperature fluctuations. The exploration of BRs will undoubtedly reach new levels once the fundamental mechanisms of homeostasis and their crosstalk with diverse phytohormones are understood. A complex regulatory network is formed by the interactions between ABA, ethylene, H_2_O_2_, and BR signaling pathways and this network affects critical processes, such as seed germination, root growth, stomatal closure, and stem elongation. These interactions enhance plant resilience to abiotic stressors, such as drought and temperature variations. Previous research has explored the mechanisms underlying BR signaling transduction pathways and the roles played by *BZR1*/*BES1* transcription factors in response to stressful conditions. Thus, the intricate pathway involving the activation of transcription factors *BZR1*/*BES1* emerges as a key determinant in BR-mediated enhancement of plant tolerance to abiotic stressors.

## Figures and Tables

**Table 1:** Role of BRs in plant tolerance to abiotic stresses.

Abiotic Stress	Plant Species	Responses	References
Cd	*Arabidopsis thaliana* L.	*Arabidopsis* root system is protected from Cd-induced stress by BRs, as they reverse its harmful morphogenic effects on apices of all root types	[41]
Low temperatures	*Lycopersicon esculentum* L.	BR-mediated enhancement of the photosynthetic apparatus and antioxidant system	[42]
Drought	*Zea mays* L.	BRs increase root and shoot growth as well as chlorophyll content, in addition to compensating for harmful drought-induced changes in maize genotypes	[43]
Water stress	*Raphanus sativus* L.	Enhanced levels of free proline, SOD, CAT, and APX, required to mitigate the repressive effects of water stress on seedlings	[44]
Drought and salinity	*Pisum sativum* L.	Increased CAT, POX, and SOD activity, leading to improved seedling growth	
Cu	*Lycopersicon esculentum* L.	Enhanced photosynthesis-related attributes and antioxidant capacity	
Cr	*Capsicum annuum* L.	EBL possesses distinct regulatory systems for mitigating Cr stress, including interactions between plant hormones, MAPK signaling, and ROS scavenging	[45]
High temperatures	*Triticum aestivum* L.	Increased CAT, POX, and SOD activity, resulting in enhanced seedling growth	[13]
Ni	*Brassica juncea* L.	Increased antioxidant enzyme activity, reducing Ni-related stress	[46]
Cu and NaCl	*Cucumis sativus* L.	Increased CAT, POX, and SOD activity, enhancing growth, carbonic anhydrase activity, and photosynthetic efficiency	[47]

## Data Availability

The data presented in this study are available on request from the corresponding author.
